# IL-17A Facilitates Platelet Function through the ERK2 Signaling Pathway in Patients with Acute Coronary Syndrome

**DOI:** 10.1371/journal.pone.0040641

**Published:** 2012-07-11

**Authors:** Shuang Zhang, Jing Yuan, Miao Yu, Hong Fan, Zhang-Qiang Guo, Rui Yang, He-Ping Guo, Yu-Hua Liao, Min Wang

**Affiliations:** 1 Laboratory of Cardiovascular Immunology, Institute of Cardiology, Union Hospital, Huazhong University of Science and Technology, Wuhan, China; 2 Department of Emergency Medicine, Union Hospital, Huazhong University of Science and Technology, Wuhan, China; 3 Department of Cardiology, Pu Ai Hospital, Tongji Medical College, Huazhong University of Science and Technology, Wuhan, China; 4 Department of Hematology, Union Hospital, Huazhong University of Science and Technology, Wuhan, China; Heart Center Munich, Germany

## Abstract

**Background:**

Platelet aggregation mediated by inflammation played a critical role in the development of coronary heart diseases (CHD). Our previous clinical researches showed that Th17 cells and their characteristic cytokine IL-17A were associated with the plaque destabilization in patients with acute coronary syndrome (ACS). However, the potent effect of IL-17A on platelets-induced atherothrombosis remains unknown.

**Methods and Results:**

In this study, we detected the plasma IL-17A levels and platelet aggregation in patients with stable angina (SA), unstable angina (UA), acute myocardial infarction (AMI) and chest pain syndrome (CPS). In addition, the markers of platelet activation (CD62P/PAC-1) and the mitogen-activated protein kinases (MAPKs) pathway were detected in platelets from ACS patients. We found that plasma IL-17A levels and platelet aggregation in patients with ACS (UA and AMI) were significantly higher than patients with SA and CPS, and the plasma IL-17A levels were positively correlated with the platelet aggregation (R = 0.47, P<0.01). In addition, in patients with ACS, the platelet aggregation, CD62P/PAC-1 and the phosphorylation of ERK2 signaling pathway were obviously elevated in platelets pre-stimulated with IL-17A in vitro. Furthermore, the specific inhibitor of ERK2 could attenuate platelet aggregation and activation triggered by IL-17A.

**Conclusion:**

Our experiment firstly proved that IL-17A could promote platelet function in patients with ACS via activating platelets ERK2 signaling pathway and may provide a novel target for antiplatelet therapies in CHD.

## Introduction

Atherosclerosis is the primary cause of cardiovascular disease among adults worldwide [1]. Platelet activation and aggregation triggered by inflammation on the ruptured atherosclerotic plaque is the critical mechanism in the development of atherosclerosis and its thrombotic complication [2]–[5].

Recently, the unique CD4^+^ Th-cell subset named Th17 cells and their characteristic cytokine IL-17A have been recognized to involve in several inflammatory diseases including experimental autoimmune myocarditis, experimental autoimmune encephalomyelitis and collagen-induced arthritis [6]–[8]. Besides, we and others have reported that IL-17A was elevated in patients with acute coronary syndrome (ACS) and atherosclerosis animal models [9], [10]. In addition, Francesco Maione etc found that IL-17A could increase the ability of platelet aggregation in healthy subjects [11]. However, the relationship between IL-17A and platelets function remains unknown in coronary heart diseases (CHD).

In our present study, we investigated the levels of plasma IL-17A and platelet aggregation, and explored whether and how IL-17A is associated with platelets function in patients with CHD.

## Materials and Methods

### Ethics Statements

This study was approved by the Ethics Committee of Tongji Medical College of Huazhong University of Science and Technology. All patients gave written informed consent and research was conformed to the guidelines of the declaration of Helsinki and its amendments.

### Patients

A total of 40 CHD patients, diagnosed by coronary angiography showing one or more coronary arteries with at least 50% stenosis, and 14 chest pain syndrome (CPS) patients were recruited from the Union Hospital, Tongji Medical College, Huazhong University of Science and Technology, Wuhan, China. All participators were divided into 4 groups: (a) In the acute myocardial infarction (AMI) group (n = 10), patients showed significant rise of serum creatine kinase MB and troponin I, ischemia symptoms, or typical elecrocardiographic changes; (b) The unstable angina (UA) group (n = 15) included patients who suffered chest pain at rest with definite ST-segment change and/or T-wave inversions; (c) In stable angina (SA) group (n = 15), patients had effort angina accompanied by downsloping or horizontal ST-segment depression>1 mm in an exercise. (d) In CPS group (n = 14), chest pain without being accompanied by electrocardiographic changes, coronary artery stenosis. Exclusion criteria were: no patients had HIV, advanced liver disease, chronic-immune-mediated diseases, renal failure, thromboembolism and infections during last four weeks. All participators were not administrated with anti-platelet, anticoagulant and anti-inflammatory drugs therapy within seven days.

### Preparation of Human Platelet Suspensions

Blood was drawn from an antecubital vein into vacutainer tube (Beckon Dickinson, California, USA) containing 3.2% sodium citrate prior to drug treatment when the patients were admitted to the hospital. Platelet-rich plasma (PRP) was prepared by centrifugation (100 g, 10 minutes, 20°C). To obtain platelet-poor plasma (PPP), the remaining blood sample was further centrifuged at 2000 g for 15 minutes. PRP was then washed twice in the presence of 100 nM prostaglandin E1 with PBS (NaCl 137 mM, KCl 2.7 mM, Na_2_HPO4 8 mM, KH_2_PO4 1.5 mM) by centrifugation at 600 g for 10 minutes. Finally, the washed platelets were suspended in HEPES-Tyrode buffer (145 mM NaCl, 5 mM KCl, 0.5 mM Na_2_ HPO_4_, 1 mM MgSO_4_, 10 mM HEPES, 5 mM D-glucose, PH 6.5). Platelet count was adjusted to 1×10^8^ platelets/L. For ELISA, blood samples were centrifuged at 2500 g for 10 minutes and plasma was stored at −80°C until analysis.

**Table 1:** Clinical characteristic of patients.

Characteristics	AMI(n = 10)	UA(n = 15)	SA(n = 15)	CPS(n = 14)
**Age (years)**	58±6	61±12	54±10	62±10
**Sex (male/female)**	7/3	10/5	11/4	8/6
**Risk factors**				
Hypertension,n (%)	7(70)	9(60)	12(80)	10(71)
Diabetes mellitus,n (%)	3(30)	3(20)	6(40)	2(14)
Smoking, n (%)	6(60)	6(40)	3(20)	2(14)
Obesity, n (%)	3(30)	5(33)	3(20)	3(21)
Hyperlipidaemia,n (%)	6(60)	6(40)	6(40)	3(20)
**Coagulant function**				
PT (s)	12.62±0.31	12.61±0.64	12.69±0.54	12.79±0.75
APTT (s)	39.16±4.39	36.76±4.18	36.81±2.09	34.19±4.57
**D-dimer(mg/L)**	0.53±0.15^**^	0.24±0.11	0.05±0.02	0.03±0.01
**hsCRP(mg/L)**	5.91±1.93^*^	3.92±1.22^*^	1.01±0.26	0.60±0.20
**Medications**				
Beta-blockers,n (%)	7(70)	7(47)	3(20)	7(50)
Nitrates, n (%)	3(30)	9(60)	9(60)	2(14)
Statins, n (%)	6(60)	9(60)	9(60)	2(14)
Calcium blockers,n (%)	3(30)	3(20)	3(20)	1(7)
ACEI, n (%)	3(30)	5(33)	9(60)	7(50)
**Left ventricular function**				
EF (%)	59.75±4.55	65.90±2.81	65.71±1.34	64.08±1.87
LVID (cm)	4.81±0.30	4.84±0.25	4.61±0.09	4.72±0.14

Data are presented as mean ± SEM or a percentage. *, p<0.05 vs SA and CPS group; **, p<0.05 vs UA, SA and CPS group.

**Figure 1:**
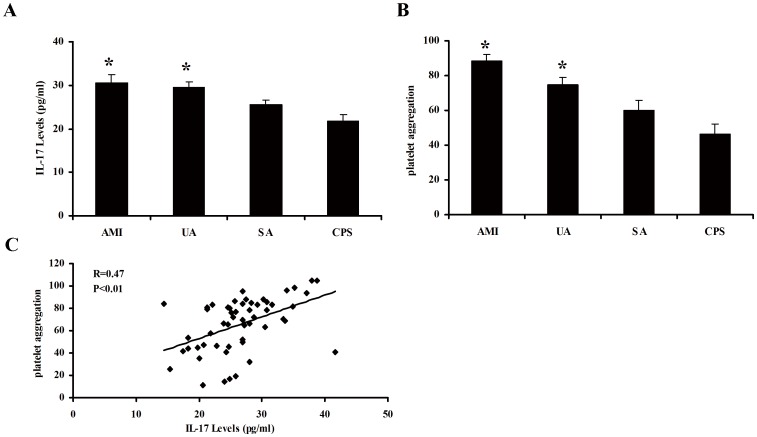
The levels of plasma IL-17A and platelet aggregation in patients with CHD. (A) The levels of plasma IL-17A were detected in different groups, including AMI (10), UA (15), SA (15) and CPS (14). (B) The levels of platelet aggregation were measured in different groups, including AMI (10), UA (15), SA (15) and CPS (14). (C) The correlation analysis of plasma IL-17A and platelet aggregation levels. Each point represents an individual patient, the correlation index (R) is 0.47, p<0.01. *, p<0.05 vs SA and CPS group. Values are means ± SEM.

### Platelet Aggregation Assay

Platelet aggregation was measured using a turbidimetric aggregation monitoring device (AggRAM, Helena Laboratories, Texas, USA). PRP was prewarmed to 37°C in a resting state. PPP was used to set the 100% aggregation. The platelet was incubated with 0.2 µg/ml IL-17A (Peprotech, Princeton, New Jersey, USA) for two minutes thereafter stimulated with adenosine diphosphate (ADP, Helena Laboratories, Texas, USA) at concentration of 3 µM. To test the effect of the inhibitors of ERK2 (U0126), p38 (SB203580) and JNK (SP600125), PRP was preincubated with the inhibitors at 10 µM for one hour. Platelet aggregation without any intervention was CTRL. Platelet aggregability was evaluated by the maximal percent of platelet aggregation.

**Figure 2:**
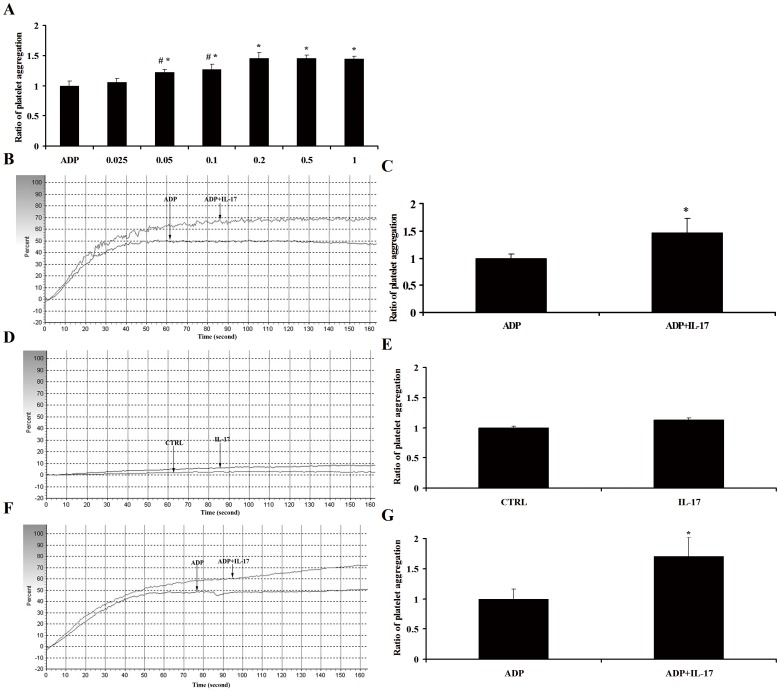
The effect of IL-17A on the platelet aggregation. (A) Platelet aggregation induced by ADP (3 µM) or ADP combined with IL-17A at 0.025, 0.05, 0.1, 0.2, 0.5 and 1 µg/ml. (B) Representative pictures for the platelet aggregation induced by ADP or ADP+IL-17A in ACS patients. (C) The results of statistical analysis for ratio of the platelet aggregation induced by ADP or ADP+IL-17A in ACS patients. (D) Representative pictures for platelet aggregation induced by IL-17A alone or CTRL (without any intervention) in ACS patients. (E) The results of statistical analysis for the ratio of platelet aggregation induced by IL-17A alone or CTRL (without any intervention) in ACS patients. (F) Representative pictures for the platelet aggregation induced by ADP or ADP+IL-17A in healthy subjects. (G) The results of statistical analysis for ratio of the platelet aggregation induced by ADP or ADP+IL-17A in healthy subjects. *, p<0.05 vs ADP group, #, p<0.05 vs 0.2 μg/ml group. Values are means ± SEM of five independent experiments with different donor platelets.

**Figure 3:**
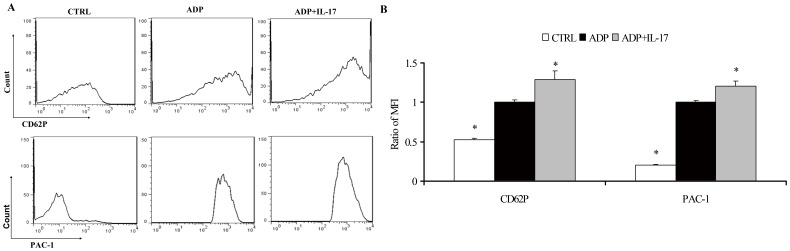
The role of IL-17A in the platelet activation. (A) Representative pictures for the CD62P and PAC-1 MFI in CTRL, ADP and ADP+IL-17A groups. (B) The results of statistical analysis for the ratio of CD62P and PAC-1 MFI in CTRL, ADP and ADP+IL-17A groups. *, p<0.05 vs ADP group. Values are means ± SEM of five independent experiments with different donor platelets.

### ELISA

Plasma levels of IL-17A assays were performed with ELISA kits (Biolegend, California, USA), according to the manufacture’s instruction. The sensitivity of IL-17A detection was 0.8 pg/ml. All samples were measured in triplicate.

**Figure 4:**
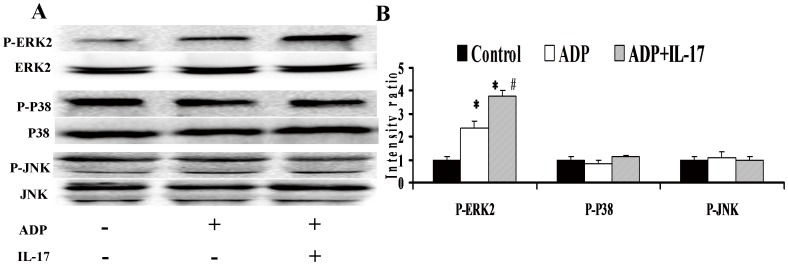
The function of IL-17A on MAPKs in the platelet. (A) Representative pictures for the MAPKs phosphorylation in different intervention. (B) The results of statistical analysis for the intensity ratio of MAPKs phosphorylation in different intervention. *, p<0.05 vs Control and ADP group; #, p<0.05 vs ADP group. Values are means ± SEM of five independent experiments with different donor platelets.

### Flow Cytometry

Washed platelets were incubated with inhibitors of mitogen-activated protein kinases (MAPKs, Cell Signaling Technology, Massachusetts, USA) including ERK2 (U0126), p38 (SB203580) and JNK (SP600125) for 1 hour followed by treated with cytokine for 2 minutes and subsequently activated with ADP for 5 minutes, and platelets without any intervention were control group (CTRL). Platelets were then incubated with APC-conjugated anti-CD61 antibody, PE-conjugated anti-CD62P antibody (Biolegend, California, USA) and FITC-conjugated PAC-1 (a confirmed monoclonal antibody that recognizes the activated integrinα_IIb_β_3_, BD Biosciences, California USA) for 30 minutes. After washing with HEPES-Tyrode buffer by centrifugation (685 g, 10 minutes), platelets were finally resuspended in 200 µl volume of HEPES-Tyrode buffer and the mean fluorescence intensity (MFI) was analyzed by FACScalibur flow cytometry (BD Biosciences, California, USA).

### Western Blot

Washed human platelets were lysed in protein lysis buffer (Pierce/Thermo Scientific, Illinois, USA) after incubation with inhibitors, IL-17A and ADP as mentioned above. Lysates were centrifuged and denatured for 10 minutes. Protein samples (30 µg) were separated by 10% SDS-polyacrylamide gel electrophoresis and electrotransferred to nitrocellulose membranes. Membranes were blocked with 5% nonfat dry milk in TBS containing 0.05% Tween 20 (TBST) at room temperature for three hours, washed three times, then incubated at 4°C overnight with different primary antibodies, including phosphor-ERK2 (1∶500, Cell Signaling Technology, Massachusetts, USA), phosphor-P38 (1∶500, Cell Signaling Technology, Massachusetts, USA), phosphor-JNK (1∶500, Cell Signaling Technology, Massachusetts, USA), ERK1/2 (1∶1000, Cell Signaling Technology, Massachusetts, USA), P38 (1∶1000, Cell Signaling Technology, Massachusetts, USA), JNK (1∶1000, Cell Signaling Technology, Massachusetts, USA). After washing three times in TBST, membranes were incubated at room temperature for two hours with HRP-conjugated secondary antibodies (1∶3000, Cell Signaling Technology, Massachusetts, USA). Membranes were washed and protein bands were visualized by using the enhanced chemiluminesence reagent (Thermo Scientific, Illinois, USA) and semiquantitatively analyzed with densitometric methods.

**Figure 5:**
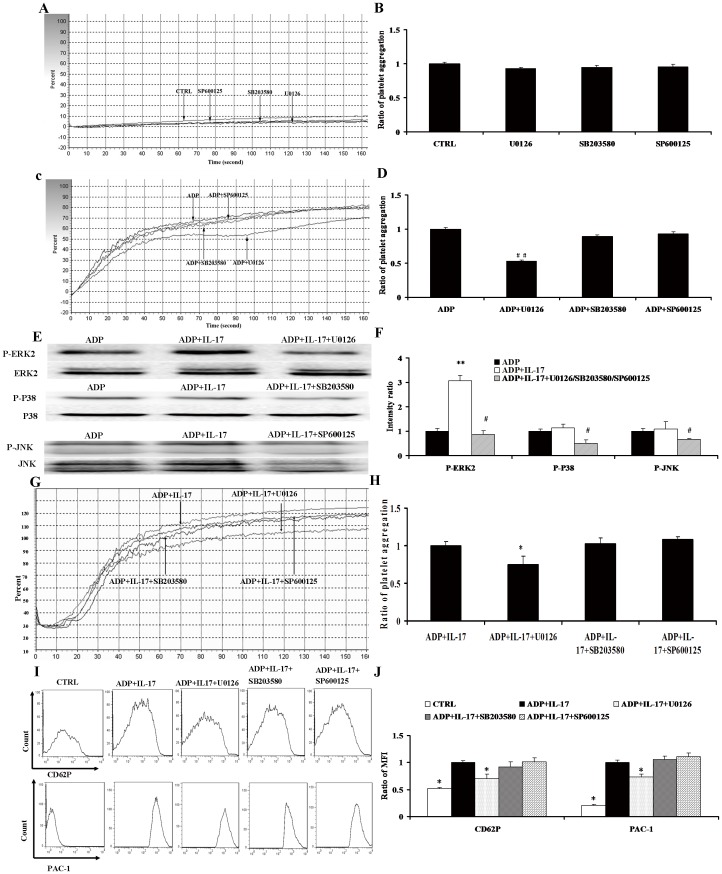
The effect of MAPKs inhibitor on platelet aggregation mediated by IL-17A combined with ADP. (A) Representative pictures for platelet aggregation response to U0126, SB203580, SP600125 and CTRL (without any intervention). (B) The results of statistical analysis for ratio of platelet aggregation response to U0126, SB203580, SP600125 and CTRL (without any intervention). (C) Representative pictures for platelet aggregation response to ADP and ADP+U0126/SB203580/SP600125. (D) The results of statistical analysis for ratio of platelet aggregation response to ADP and ADP+U0126/SB203580/SP600125. (E) The levels of MAPKs phosphorylation were measured using Western blot analysis in ADP, ADP+IL-17A and ADP+IL-17A+U0126/SB203580/SP600125 group. (F) The results of statistical analysis for the MAPKs phosphorylation in ADP, ADP+IL-17A and ADP+IL-17A+U0126/SB203580/SP600125 group. (G) Representative pictures for the platelet aggregation in ADP+IL-17A and ADP+IL-17A+U0126/SB203580/SP600125 group. (H) The results of statistical analysis for the ratio of platelet aggregation in ADP+IL-17A and ADP+IL-17A+U0126/SB203580/SP600125 group. (I) Representative pictures for the CD62P and PAC-1 MFI in CTRL (without any intervention), ADP, ADP+IL-17A and ADP+IL-17A+U0126/SB203580/SP600125 group. (J) The results of statistical analysis for the ratio of CD62P and PAC-1 MFI in CTRL (without any intervention), ADP, ADP+IL-17A, and ADP+IL-17A+U0126/SB203580/SP600125 group. *, p<0.05 vs ADP+IL-17A and ADP+IL-17A+SB203580/SP600125 group; **,p<0.05 vs ADP group. #, p<0.05 vs ADP+IL-17A group. Values are means ± SEM. ##, p<0.05, vs ADP and ADP+SB203580/SP600125 group.

### Statistical Analysis

Data were shown as the mean ± SEM. Statistical analysis of the data was performed with 2-tailed Student’s t test (two groups) or one-way ANOVA (more than two groups), and the correlation between two variables was tested by bivariate correlation analysis using SPSS11.0. P<0.05 was considered statistically significant.

## Results

### Clinical Characteristics of Patients

In patients with AMI and UA, the levels of plasma hsCRP were higher than patients with SA and CPS (p<0.05), and there was no obvious change in plasma hsCRP between the AMI and UA patients. In addition, the levels of plasma D-dimer were elevated markedly in patients with AMI compared with those in UA, SA and CPS patients (p<0.05), and no significant difference was found among UA, SA and CPS patients (Table 1).

### The Levels of Plasma IL-17A and Platelet Aggregation in Patient with CHD

The levels of plasma IL-17A and platelet aggregation were elevated markedly in patients with AMI and UA compared with SA and CPS (all p<0.05, Fig. 1A–B), and there was no significant difference between the SA and CPS group. In addition, the levels of plasma IL-17A were positively correlated with the levels of platelet aggregation (R = 0.47, p<0.01, Fig. 1C).

### The effect of IL-17A on the Platelet Aggregation

To investigate the direct role of IL-17 in platelet aggregation, we prepared the platelet suspensions in ACS patients and performed the experiment of platelet aggregation in vitro. Firstly, we explored the optimal concentration of IL-17A on platelet aggregation, and found that the effect of IL-17A on ADP-induced platelet aggregation was concentration-dependent, with detectable effect at 0.05 µg/ml and reaching maximum at 0.2 µg/ml (Fig. 2A). Subsequently, we confirmed that IL-17A could dramatically enhance platelet aggregation when synergized with ADP (Fig. 2B–C), while it alone did not influence platelet aggregation (Fig. 2D–E). Additionally, we also demonstrated the facilitative effect of IL-17A on ADP-induced platelet aggregation in healthy subjects (Fig. 2F–G).

### The Role of IL-17A in the Platelet Activation

To elucidate the direct activation effect of IL-17 on platelets, we detected the activation markers (CD62P and PAC-1) on the membrane of platelets, and the data were analyzed by the ratio of the MFI compared with the platelets stimulated with ADP alone. The ratio levels of the MFI for CD62P and PAC-1 in ACS patients were much higher in platelets pre-incubated with IL-17A in addition of ADP than those stimulated with ADP alone (p<0.05, Fig. 3A–B).

### The Function of IL-17A on MAPKs in the Platelet

The phosphorylation levels of ERK2 in ACS patients were increased dramatically in platelets treated with IL-17A and ADP, compared with those in platelets stimulated with ADP alone. No obviously difference was observed in phosphorylation levels of p38 and JNK in each treatment group (p<0.05, Fig. 4A–B).

### The Effect of MAPKs Inhibitor on Platelet Aggregation Mediated by IL-17A Combined with ADP

In order to rule out the effect of MAPKs inhibitors on platelet aggregation, we firstly detected platelet aggregation induced by U0126 (ERK2), SB203580 (p38), SP600125 (JNK), respectively. The result demonstrated that these inhibitors did not influence platelet aggregation (Fig. 5A–B). Then we measured the effect of the inhibitors on ADP-induced platelet aggregation, which indicated that ADP-induced platelet aggregation was markedly suppressed by U0126, compared with SB203580 and SP600125 (Fig. 5C–D) the effect of the inhibitors on t aggregation regation. To further elucidate the inhibition effect of the inhibitors on platelet function induced by IL-17 and ADP, platelets preincubated with IL-17/ADP were treated with these specific kinase inhibitors. Although the phosphorylation of ERK, p38, and JNK was obviously inhibited by the corresponding inhibitors (Fig. 5E–F), platelet aggregation and the CD62P/PAC-1 expression were dramatically surppressed in IL-17A+ADP+U0126 group compared with IL-17A+ADP group and IL-17A+ADP+SB203580/SP600125 group (all p<0.05, Fig. 5G–J). There was no significant change in platelet aggregation and the CD62P/PAC-1 expression between IL-17A+ADP group and IL-17A+ADP+SB203580/SP600125 group.

## Discussion

Coronary heart disease is a progressive inflammatory disease characterized by the formation of plaque, plaque rupture, and ultimately atherothrombosis [12]. Accumulative evidences gathered recently proved that IL-17A, the proinflammatory cytokine, might serve as a proatherogenic agent in coronary heart disease [9], [10], [13]–[15]. However, inhibition of IL-17A could not restrict lesion development [16]. Thus, the role of IL-17A in atherosclerosis remained controversial [17]. Actually, these studies focused on the role of IL-17A in the formation of atherosclerotic plaque, the potent effect of IL-17A on platelets-induced atherothrombosis needed further investigation.

In our experiment, we measured plasma IL-17A levels and platelet aggregation in patients with CPS, SA and ACS (including UA and AMI). The results revealed that plasma IL-17A levels and platelet aggregation were markedly higher in patients with ACS than those with SA and CPS. Moreover, plasma IL-17A concentrations were positively correlated with platelet aggregation. These phenomenons suggested that IL-17A might promote platelet aggregation in ACS patients. In order to mimic the pathological environment in vivo, we subsequently performed the platelet aggregation experiment with pathogenetic doses of IL-17A in vitro, and found that IL-17A alone did not influence platelet aggregation while it could significant enhance platelet aggregation in ACS patients when combined with ADP. Because the large doses of IL-17A were used, the facilitative effect of IL-17A on ADP-induced platelet aggregation was also observed in healthy subjects. These data indicated that IL-17A might serve as a synergetic agent augmenting ADP-induced platelet aggregation, which was mainly due to controlling the stability of target genes mRNA transcripts via MAPKs signaling pathway induced by IL-17A [18].

Platelet aggregation based on platelet activation, especially the membrane expression of CD62P and exposure of α_IIb_β_3_ integrin recognized by the monoclonal antiboby PAC-1. In our study, we proved that IL-17A could promote the exposure of α_IIb_β_3_ integrin, which provided more ligand binding site for fibrinogen via conformational change and crosslinked the neighboring activated platelets and resulted in platelet aggregation [4], [5]. In addition, enhanced expression of CD62P induced by IL-17A could interact with the P-selectin glycoprotein ligand 1 receptor expressed on monocytes and mediate the rolling of monocytes on activated endothelium, which facilitated platelet-leukocyte aggregation and induced the formation of platelet thrombus [3], [4], [19]. Furthermore, in parallel with CD62P exposure, the α-granule contents such as fibrinogen, fibronectin and von Willebrand factor were also released from the activation of platelets. These contents could further facilitate platelet adhesive and aggregation [5]. Thus, we concluded that IL-17A could evidently augment ADP-induced platelet activation mediated by CD62P and α_IIb_β_3_ integrin, resulting in the platelet aggregation and atherothrombosis.

IL-17A receptor is extensively expressed in a variety of tissues as well as in peripheral blood cells [20]. The specific binding of IL-17A and its receptor could induce the activation of NF-κB and MAPKs including ERK2, p38 and JNK1 [20]. In platelets, MAPKs mainly contributed to platelet activation and aggregation induced by various stimuli, such as ADP, thrombin and collagen [21]. To gain insight into the mechanism of IL-17A in platelets activation, we investigated the effect of IL-17A on MAPKs signaling in platelets. Our result showed that IL-17A enhanced ERK2 phosphorylation rather than p38 and JNK1 phosphorylation when combined with ADP. The potentially mechanism might be that ADP bound to Gq-coupled P2Y1 receptor and Gi-coupled P2Y12 receptor which in turn activated Src kinase, resulting in the activation of ERK2 [22] Simultaneously, IL-17A could also activate ERK2 by its receptor via a proximal adaptor Act1 [18]. Thus, IL-17A combined with ADP could significantly enhance the activation of ERK2 through different signaling intermediates Act1 and Gq/Gi.

To further define the effects of IL-17A on MAPks pathway in platelets, the ERK2, p38, and JNK1 inhibitors were administrated respectively. As expected, the phosphorylation of ERK, p38 and JNK was markedly blocked by the specific kinase inhibitors. The ERK2 inhibitor U0126 caused a strongly inhibition of aggregation and activation of platelets induced by ADP combined with IL-17A. However, p38 and JNK inhibitors didn’t influence the aggregation and activation of platelets induced by ADP and IL-17A. In addition, the inhibitors alone did not influence platelet aggregation, and only U0126 could obviously inhibit the level of ADP-induced platelet aggregation. Overall, we concluded that U0126 not only inhibits ADP-induced platelet aggregation but also the stimulatory effect of IL-17A on ADP induced aggregation, which further confirmed the importance of ERK2 signaling pathway in platelet function costimulated with IL-17A and ADP.

Although we have proved the effect of IL-17A on platelet function in different aspects, the limitations of our study still exist. The IL-17A receptor antagonist or IL-17A receptor knockout mice should be used to provide a more direct evidence for the pro-aggregant role of IL-17A. Besides, we need to explore the synergetic mechanism of IL-17A on platelet function. In spite of this, our researches still have clinical significance. We firstly revealed that IL-17A was highly effective in promoting platelet activation and aggregation in ACS patients through the ERK2 signaling pathway. It gives us a suggestion that blocking of IL-17A in ACS patients may be a promising therapeutic method in reducing the formation of atherothrombosis in future.

## References

[1] Anderson GF, Chu E (2007). Expanding priorities – confronting chronic disease in countries with low income.. N Engl J Med 356.

[2] Hansson GK, Libby P, Schonbeck U, Yan ZQ (2002). Innate and adaptive immunity in the pathogenesis of atherosclerosis.. Cir Res.

[3] Davi G, Patrono C (2007). Platelet activation and atherothrombosis.. N Engl J Med 357.

[4] Wagner DD, Burger PC (2003). Platelets in inflammation and thrombosis.. Arterioscler Thromb Vasc Biol.

[5] Ruggeri ZM (2002). Platelets in atherothrombosis.. Nat Med.

[6] Lubberts E (2010). Th17 cytokines and arthritis.. Semin Immunopathol.

[7] Yuan J, Yu M, Lin QW, Cao AL, Yu X (2010). Th17 cells contribute to viral replication in coxsackievirus B3-induced acute viral myocarditis.. J immunol.

[8] Noubade R, Krementsov DN, Del Rio R, Thornton T, Nagaleekar V (2011). Activation of p38 MAPK in CD4 T cells controls IL-17 production and autoimmune encephalomyelitis.. Blood.

[9] Cheng X, Yu X, Ding YJ, Fu QQ, Xie JJ (2008). The Th17/Treg imbalance in patients with acute coronary syndrome.. Clin Immunol.

[10] Xie JJ, Wang J, Tang TT, Chen J, Gao XL (2010). The Th17/Treg functional imbalance during atherogenesis in ApoE^−/−^ mice.. Cytokine.

[11] Maione F, Cicala C, Liverani E, Mascolo N, Perretti M (2011). IL-17A increases ADP-induced platelet aggregation.. Biochem Biophy Res Commun.

[12] Libby P (2002). Inflammation in atherosclerosis.. Nature.

[13] Erbel C, Chen L, Bea F, Wangler S, Celik S (2009). Inhibition of IL-17A attenuate atherosclerotic lesion development in ApoE-Deficient Mice.. J Immunol.

[14] Von vietinghoff S, Ley K (2010). Interleukin 17 in vascular inflammation.. Cytokine Growth Factor.

[15] Smith E, Prasad KM, Butcher M, Dobrian A, Kolls JK (2010). Blockade of Interleukin-17A Results in Reduced Atherosclerosis in Apolipoprotein E-Deficient Mice.. Circulation.

[16] Cheng X, Taleb S, Wang J, Tang TT, Chen J (2011). Inhibition of IL-17A in atherosclerosis.. Atherosclerosis.

[17] Tableb S, Tedqui A, Mallat Z (2010). Interleukin-17: friend or foe in atherosclerosis?. Curr opin Lipidol.

[18] Gaffen SL (2009). Structure and signaling in the IL-17 receptor family.. Nat Rev Immunol.

[19] Burger PC, Wagner DD (2003). Platelet P-selectin facilitate atherosclerotic lesion development.. Blood.

[20] Moseley TA, Haudenschild DR, Rose L, Reddi AH (2003). Interleukin-17 family and IL-17 receptors.. Cytokine Growth Factor Rev.

[21] Adam F, Kauskot A, Rosa JP, Bryckaert M (2008). Mitogen-activated protein kinases in hemostasis and thrombosis.. J Thromb Haemost.

[22] Garcia A, Shankar H, Muruqappan S, Kim S, Kunapuli SP (2007). Regulation and functional consequences of ADP receptor-mediated ERK2 activation in platelets.. Biochem J.

